# The Chaperone ClpX Stimulates Expression of *Staphylococcus aureus* Protein A by Rot Dependent and Independent Pathways

**DOI:** 10.1371/journal.pone.0012752

**Published:** 2010-09-14

**Authors:** Lotte Jelsbak, Hanne Ingmer, Lukás Valihrach, Marianne Thorup Cohn, Mie H. G. Christiansen, Birgitte H. Kallipolitis, Dorte Frees

**Affiliations:** 1 Department of Veterinary Disease Biology, Faculty of Life Sciences, University of Copenhagen, Frederiksberg C, Denmark; 2 Department of Biochemistry and Molecular Biology, University of Southern Denmark, Odense, Denmark; National Institute of Allergy and Infectious Diseases, National Institutes of Health, United States of America

## Abstract

The Clp ATPases (Hsp100) constitute a family of closely related proteins that have protein reactivating and remodelling activities typical of molecular chaperones. In *Staphylococcus aureus* the ClpX chaperone is essential for virulence and for transcription of *spa* encoding Protein A. The present study was undertaken to elucidate the mechanism by which ClpX stimulates expression of Protein A. For this purpose, we prepared antibodies directed against Rot, an activator of *spa* transcription, and demonstrated that cells devoid of ClpX contain three-fold less Rot than wild-type cells. By varying Rot expression from an inducible promoter we showed that expression of Protein A requires a threshold level of Rot. In the absence of ClpX the Rot content is reduced below this threshold level, hence, explaining the substantially reduced Protein A expression in the *clpX* mutant. Experiments addressed at pinpointing the role of ClpX in Rot synthesis revealed that ClpX is required for translation of Rot. Interestingly, translation of the *spa* mRNA was, like the *rot* mRNA, enhanced by ClpX. These data demonstrate that ClpX performs dual roles in regulating Protein A expression, as ClpX stimulates transcription of *spa* by enhancing translation of Rot, and that ClpX additionally is required for full translation of the *spa* mRNA. The current findings emphasize that ClpX has a central role in fine-tuning virulence regulation in *S. aureus*.

## Introduction

*Staphylococcus aureus* is an opportunistic pathogen capable of causing a variety of diseases in humans, ranging from localized infections of skin and soft tissue to life-threatening systemic infections [1]. The pathogenicity of *S. aureus* relies on a wide array of surface-bound and secreted virulence factors that provide the bacterium with the ability of tissue binding, tissue destruction, and immune evasion [2]. These virulence factors are coordinately produced in a growth phase dependent manner. The cell-surface associated factors are primarily expressed during exponential growth phase, whereas expression of the secreted factors is induced upon transition to stationary phase. Central for this regulation is the quorum sensing *agr* locus [3].

Protein A is a major surface bound virulence factor found in all examined strains of *S. aureus* [4]. It is well-known for its ability to bind the Fc-region of IgG from several mammalian species [5]. Additionally, Protein A can bind von Willebrand factor, and is capable of inducing inflammatory responses in the host [5,6]. Accordingly, the importance of Protein A in infections has been demonstrated in several animal models [7–9].

**Figure 1:**
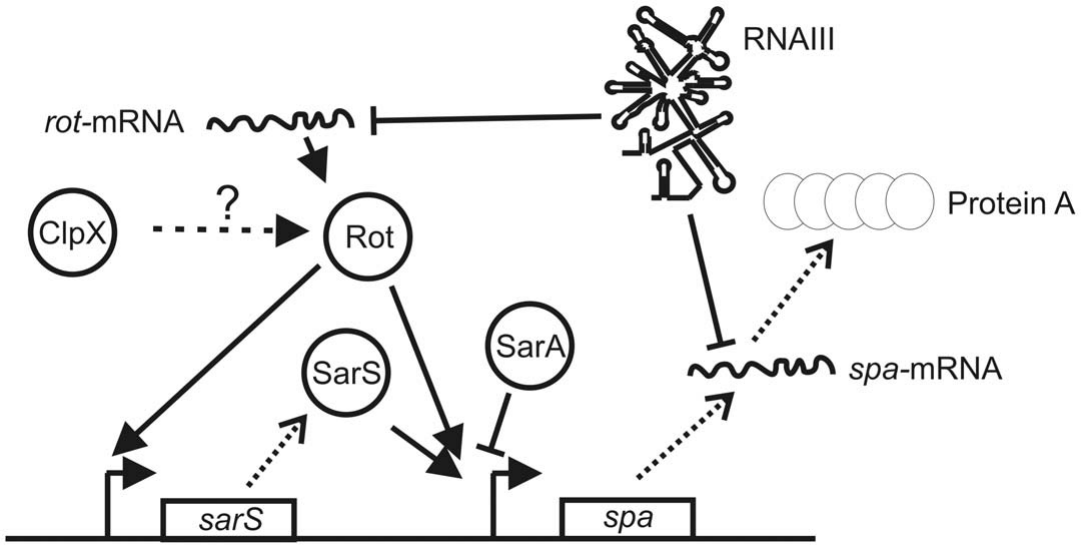
Model depicting the regulatory network controlling Protein A expression. Transcription of *spa* is positively regulated by the transcriptional activators Rot and SarS and negatively regulated by SarA [10,11,13,41]. Translation of *spa* and *rot* mRNAs is inhibited by RNAIII [12]. We hypothesized that ClpX stimulates Protein A expression by stimulating either synthesis or activity of Rot (indicated by the dotted arrow). See text for further details. Solid arrows indicate activation, while solid T-bars indicate repression. Proteins are indicated by spheres. mRNAs are indicated by wavy lines. Promoters are indicated by bent arrows.

Expression of Protein A is regulated by growth phase and is controlled by complex regulatory networks acting at both the transcriptional, translational and post-translational levels [10–13]. The complex regulation of Protein A expression has been schematically depicted in Fig. 1. At the pinnacle of this regulatory network is the Agr quorum sensing system reviewed in [14]. The effector molecule of the *agr* quorum sensing system is RNAIII, a small regulatory RNA that is strongly induced in the post-exponential growth phase [14]. RNAIII is 514 nucleotides long and folds into a complex secondary structure comprising 14 distinct stem-loops [15]. Recent evidence supports that RNAIII fulfils its role as a global virulence regulator primarily by controlling translation of target genes [12,16–18]. In regard to Protein A expression, RNAIII acts both directly and indirectly to reduce synthesis of the protein. Directly, RNAIII down regulates expression of Protein A by binding to the *spa* mRNA [12]. This binding reduces Protein A synthesis at two levels, as it both inhibits translation and promotes degradation of the *spa* mRNA [12]. Indirectly, RNAIII reduces transcription of *spa* by inhibiting translation of Rot [17]. As illustrated in Fig. 1, Rot activates *spa* transcription directly by binding to the *spa* promoter, and additionally, by enhancing transcription of *sarS*, encoded directly upstream of *spa* [11,13]. Similar to Rot, SarS activates *spa* transcription through direct interactions with the *spa* promoter region [10,11,13,19]. The activator activities of Rot and SarS are counteracted by the repressor SarA [10,13]. Present models suggest that SarS and Rot act synergistically to promote *spa* transcription, whereas the repressor SarA and the activator SarS compete for overlapping binding sites in the *spa* promoter [10,11,13].

We recently revealed an additional layer of regulation by showing that the levels of *spa* transcript and Protein A were severely reduced in mutants lacking ClpX [20,21]. Notably, we additionally showed that the *clpX* gene is essential for virulence of *S. aureus* [21]. The ClpX protein belongs to the family of closely related Clp ATPases that function as molecular chaperones [22,23]. Additionally, ClpX can interact with the unrelated ClpP peptidase forming a proteolytic complex [24–26]. In the ClpXP protease, ClpX serves to specifically recognize and subsequently unfold and translocate the substrate into the ClpP proteolytic chamber for degradation [24,26–28]. Interestingly, we found that Protein A expression was un-affected by the absence of ClpP [20,21]. We therefore hypothesized that it is the ClpP independent chaperone activity of ClpX that is required for synthesis of Protein A. Further genetic analyses indicated that ClpX stimulates *spa* transcription by controlling either activity or synthesis of the transcriptional regulator Rot (Fig. 1) [20,21]. The present study was undertaken to elucidate the molecular mechanism by which ClpX regulates Protein A expression. Here, we report that expression of Protein A requires a threshold level of Rot. In the absence of ClpX, the Rot content is below this threshold level resulting in lack of Protein A expression. An important finding is that ClpX enhances expression of Rot at the level of translation, and that ClpX additionally enhances translation of the *spa* mRNA. Hence, ClpX performs dual roles in regulating Protein A expression, as ClpX stimulates transcription of *spa* indirectly by enhancing translation of Rot and additionally enhances translation of the *spa* mRNA.

## Materials and Methods

### Bacterial strains and growth conditions

Bacterial strains and plasmids are listed in Table 1. *S. aureus* 8325-4 and SA564 were used as wild-type strains. As primary recipient for plasmids the restriction deficient strain *S. aureus* RN4220 [29,30] was used. *S. aureus* strains were grown in Tryptic Soya Broth media (TSB; Oxoid) under vigorous agitation (200 rpm) at 37°C. Usually 20 ml of medium was inoculated in 200 ml flasks to allow efficient aeration of the medium. For solid medium, 1.5% agar was added to give TSA plates. Erythromycin (5 µg ml^−1^), chloramphenicol (5 µg ml^−1^), or tetracycline (5 µg ml^−1^) was added as required. Upon receipt of the low-passage isolate SA564 (from G. Somerville) the strain was cultured once and stored frozen at −80°C. In all experiments we used SA564 streaked freshly from this stock. *Escherichia coli* strains Top10, Top10F' or StrataClone Solopack Competent Cells were used for DNA cloning and were grown in Luria–Bertani (LB; Oxoid) broth or on LB agar plates at 37°C. Tetracycline (10 µg ml^−1^), kanamycin (50 µg ml^−1^), chloramphenicol (35 µg ml^−1^) or ampicillin (50 µg ml^−1^) was added as required.

**Table 1:** Bacterial strains and plasmids used.

S. aureus strains	Relevant genotype	Reference
8325-4	Reference strain	[29]
RN4220	Restriction-deficient mutant of 8325-4	[30]
RN6911	8325-4, *agr*::*tetM* (Tet^R^)	[46]
HI2209	8325-4:: Δ*clpX*	[21]
WA525	8325-4, *rot*::*erm*	[11]
HI2433	8325-4, rot::TN917	[47]
DF2269	8325-4:: Δ*clpX*, *agr::tetM* (Tet^R^)	[20]
LJ93	8325-4/pLOJ132, (Erm^R^)	This work.
LJ97	agr/pLOJ132, (Erm^R^) (Tet^R^)	This work.
LJ94	Δ*clpX*/pLOJ132, (Erm^R^)	This work.
LJ135	Δ*clpX*; *agr*/pLOJ132, (Erm^R^) (Tet^R^)	This work.
LJ98	8325-4/pLOJ133, (Erm^R^)	This work.
LJ102	*agr*/pLOJ133, (Erm^R^) (Tet^R^)	This work.
LJ99	Δ*clpX*/pLOJ133, (Erm^R^)	This work.
LJ133	Δ*clpX*; agr/pLOJ133, (Erm^R^) (Tet^R^)	This work.
LJ113	8325-4/pLUG520, (Erm^R^)	[12]
LJ117	agr/pLUG520, (Erm^R^) (Tet^R^)	[12]
LJ115	Δ*clpX*/pLUG520, (Erm^R^)	This work.
LJ121	Δ*clpX*; *agr*/pLUG520, (Erm^R^) (Tet^R^)	This work.
HI2438	8325-4, hfq mutant (Cam^R^)	This work.
LJ118	WA525/pWA163.	This work.
SA564	Clinical isolate	[37]
HI2781	SA564:: Δ*clpX*	This work.
HI2870	8325-4:: Δ*clpX+clpX*	This work.
HI2871	SA564:: Δ*clpX+clpX*	This work.
**E. coli strains**		
Top10	Cloning strain	Invitrogen
Top10F'	Cloning strain	Invitrogen
ER2566	Protein expression strain	NEB
Strataclone cells	Cloning strain	Stratagene
**Plasmids**		
pET28a^+^	His-tag vector (Kan^R^)	Novagen
pET100/D	His-tag vector (Amp^R^)	Invitrogen
pRMC2	shuttle-vector with tet-inducible promoter (Cam^R^)	[32]
pLOJ104	pET28a^+^ derivative with *rot* -*his_6_*, (Kan^R^)	This work.
pET100clpX	pET100/D-derivative with *clpX*-*his_6_*, (Amp^R^)	This work
pLUG520	PrpoB:spa *lacZ* translational fusion, (Erm^R^)	[12]
pLOJ132	P*rpoB*::*rot lacZ* translational fusion, (Erm^R^)	This work
pLOJ133	P*rpoB*:: *lacZ* transcriptional fusion, (Erm^R^)	This work
pUC19	*E. coli* cloning vector (Amp^R^)	This work
pLOJ131	P*rpoB*:*rot lacZ* fusion in pUC19 (Amp^R^)	This work
pWA163	P*xylA*:*rot* (Tet^R^)	[11]
pASrot	P*tetO*::*rot* (anti-sense) (Cam^R^)	This work

### Plasmid and strain construction

#### SA564*ΔclpX*

For construction of SA564*ΔclpX*, pSaΔclpX from RN4220 [21] was electroporated into competent SA564 cells at 30°C as described in [31]. A strain containing a 655 bp in frame deletion in the *clpX* gene was obtained by allelic replacement as described in [21], except that the final plasmid-loss was performed at 37°C. Verification of the chromosomal *clpX* deletion was done as described in [21].

### Restoration of the chromosomal clpX locus in 8325-4ΔclpX and SA564 ΔclpX

As we were unable to clone an error-less *clpX* gene in *E. coli* an alternative strategy was employed to complement the *clpX* deletion in 8325-4 and SA564:

In the course of attempting to create a *clpX* deletion in the strain Newman following the same procedure as described in the present manuscript and in [21], we obtained the desired chromosomal deletion of *clpX* (D. Frees, unpublished data). However, following replacement recombination these colonies still maintained a plasmid-borne copy of the intact *clpX* gene (and its promoter). This plasmid was transformed into SA564 and 8325-4, and used to restore the chromosomal *clpX* locus by doing the same double cross-over procedure as initially used to create the *clpX* deletion [21]. Colonies containing an intact chromosomal copy of the *clpX* gene were identified by colony PCR using the primers SaclpX385f (5′-GACGATGCAGAACAACGTG) and SaclpX2447r (5′-CCATACCCTGGAACATCCAC), and subsequently plasmid loss was achieved by growing at the non-permissive temperature.

### Construction of pASrot expressing anti-sense *rot* from an inducible promoter

To vary the Rot content in SA564 we used the expression vector pRMC2 [32]. This plasmid harbors an improved copy of the *tetO* promoter in pALC2073 that ensures complete repression when non-induced and high expression when induced by anhydrotetracycline. The *rot* gene was PCR-amplified from 8325-4 chromosomal DNA using the primers KpnI-rotF (5′-TTACCATGGTACC ATTGGGAGATGTTTAGCATG) and SacI-rotR (5′-ACATCAGAGCTC CCAACGTAATCATGCTCCAT) which, except for the added restriction sites (underlined), are the same primers that J. Oscarsson used to clone *rot i*n pWA163 [11]. The PCR-fragment was TOPO-cloned (Invitrogen), and after confirming that the resulting plasmid contained an error-less *rot*-gene, it was cloned into pRMC2 in the anti-sense orientation using *Sac*I and *EcoR*I. The plasmid was first introduced into RN4220, purified from this strain using the Qiagen Plasmid Midi prep kit and then introduced into SA564 by electroporation as described by [31].

#### pLOJ132

A fragment containing the *rpoB*-promoter region, the first part of the *rpoB*-leader, but leaving out the Shine-Dalgarno and startcodon was PCR amplified using the primers Prpofwd (5′-AATGAATTCGTAAAGGAAAGTGATGC-3′) and PrpoRevNy (5′-TCAGGTACCAAAAATTATGTGATCCGCTTTTAGC-3′), digested with *EcoR*I and *Kpn*I and inserted into pUC19 generating pLOJ130. A fragment containing part of the *rot*-leader [33] and the first 3 aa codons was PCR amplified using the primers o-loj-rotmfwdny (5′-GCGGTACCTTATGCATAAGTTAGCACATAC-3′) and o-loj-rotmrev (5′-GCGGA TCCTTTTTTCATGCTAAACATC-3′), digested with *Kpn*I and *BamH*I and inserted into pLOJ130 generating pLOJ131. Subsequently, an *EcoR*I—*BamH*I-fragment from pLOJ131containing the P*rpoB*-*rot*-leader was gel purified (GFX) and ligated into pLUG520 where the P*rpoB*-*spa*-fragment had been excised using the same enzymes. This resulted in pLOJ132; P*rpoB*-*rot*-leader translationally fused to *lacZ* in a pLUG520 derivative.

#### pLOJ133

A fragment containing the *rpoB*-promoter region, the *rpoB*-leader, and the Shine-Dalgarno and startcodon was PCR amplified using the primers Prpofwd and PrpoRev2 (5′-GCGGATCCTGCCAAACAGATTCAC-3′), digested with *EcoR*I and *BamH*I and ligated into pLUG520 where the P*rpoB*-*spa*-fragment had been removed using the same enzymes. This resulted in pLOJ133; P*rpoB* translationally fused to *lacZ* in a pLUG520 derivative.

The plasmids were introduced into *S. aureus* strain RN4220 by electroporation and purified using the miniprep kit from Omega. The plasmid isolation procedure was modified by incubating the cell suspension in solution I containing 50 µg ml^−1^ lysostaphin (Sigma) for 1 h at 37°C. The purified plasmids were subsequently introduced into other *S. aureus* strains by electroporation. Transductions were performed as described by [34] using *ϕ*11.

### Overexpression and purification of Rot and ClpX His-tagged fusion proteins and generation of anti-Rot- and anti-ClpX antibodies

A fragment containing the full-length *rot* gene was generated by PCR using primers o-loj-rot-7 (5′-GGATTTCCATATGAAAAAAGTAAATAACGACACTG-3′) and o-loj-rot-10 (5′-GGGAATTCTTACACAGCAATAATTCGGTTTAAACTATTTTGC-3′), digested with *Nde*I and *EcoR*I and cloned in-frame with six N-terminal histidine codons downstream of the IPTG-inducible T7 promoter of pET28a+ (Novagen) resulting in plasmid pLOJ104. DNA sequence analysis was performed to verify the construction and the absence of mutations. However, we were unable to recover error-less clones and for the purpose of antibody production we chose to continue with a clone containing a mutation leading to an Ala129→Arg substitution.

A fragment containing the full-length *clpX* gene was generated by PCR using primers ClpX100fwd (5′-caccATGTTTAAATTCAATGAAGATGAAGAAAATTTG-3′) and ClpX100rev (5′-TTA AGCTGATGTTTTACTATTATTAATTAAATTGCC-3′), TOPO-cloned into pET100D/TOPO (Invitrogen) in-frame with six N-terminal histidine codons downstream of the IPTG-inducible T7 promoter resulting in plasmid pET100clpX. DNA sequence analysis was performed to verify the construction and the absence of mutations. However, we were unable to recover error-less clones and for the purpose of antibody production we chose to continue with a clone containing a single amino substitution.

To induce synthesis of the His-tagged Rot and ClpX proteins in *E. coli*, ER2566/pLOJ104 and ER2566/pET100clpX were grown in 2xLB to an OD_600_ of 1.0 and then induced by adding IPTG to a final concentration of 1.0 mM for 2 h. The overexpressed proteins were purified on a Nickel NTA resin as described by the manufacturer (Qiagen) and 200 µg of pure protein was used to immunize a rabbit (antibodies were produced by CovalAB Company) using standard procedures (Sambrook et al., 1989).

### Overexpression and purification of Hfq protein and generation of anti-Hfq-antibodies

For purification of Hfq, the intein system (Impact-CN; New England Biolabs) was used. Protein purification was performed as described previously [35]. Anti-Hfq-antibody was produced by Charles River Laboratories by immunizing rabbits with purified Hfq protein. The specificity of the antibody was tested by Western blot analysis on purified Hfq protein and protein extracts.

### Western blotting

*S. aureus* strains were grown in TSB as specified above. Exponential cells were harvested at OD_600_ = 1.0 (+/−0.1), and post-exponential samples at OD_600_ = 2.0 (+/−0.1), or OD_600_ = 3.0 (+/−0.1), and used for isolation of intracellular proteins. One ml aliquots were harvested and kept at −80°C until all samples were collected. The cell pellets were thawed on ice, resuspended in 50 mM TrisHCl, Ph = 7.4 to a calculated OD_600_ of 10.0. PMSF, Dnase, Rnase and lysostaphin were added to the samples, and they were incubated at room-temperature for 15 min. Cellular debris was removed by centrifugation and the protein concentration of each sample was measured using the Bradford dye-binding procedure from Bio-Rad. For immunoblotting a total of 5 µg (or 10 µg for Hfq-immunoblotting) of each sample was loaded on NuPAGE® 4–12% Bis-Tris gels (Invitrogen) using MOPS-Buffer (Invitrogen). The proteins were blotted onto a polyvinylidene difluoride membrane using the XCell II blotting module (Invitrogen). The membranes were pre-blocked with IgG. The Rot and ClpX proteins were probed using Rabbit-anti-Rot-antibody or Rabbit-anti-ClpX-antibody at a 1∶2000 dilution. Protein A was probed using monoclonal Mouse-anti-Protein-A-antibodies (Sigma) at a 1∶10000 dilution. The Hfq protein was probed using anti-Hfq antibodies at a 1∶2500 dilution. Bound antibody was detected with the WesternBreeze Chemiluminescent Anti-Rabbit kit or Anti-mouse kit (Invitrogen). All Western blots were repeated at least three times with similar results.

### RNA extraction and Northern blot analysis

Cells were grown in TSB at 37°C with shaking to the indicated OD_600_. 1 ml aliquots were harvested and immediately frozen and stored at -80°C. Cells were lysed mechanically using the FastPrep system (Bio101; Q-biogene), and RNA was isolated using the RNeasy mini kit (QIAGEN, Valencia, Calif.) according to the manufacturer's instructions. Total RNA was quantified by spectrophotometric analysis (λ = 260 nm), and 5 µg of RNA of each preparation was loaded onto a 1% agarose gel and separated in 10 mM sodium phosphate buffer as described previously [20]. RNA was transferred to a positively charged nylon membrane (Boehringer Mannheim) by capillary blotting as described by Sambrook et al. Hybridization was performed according to [20] using gene-specific probes that had been labeled with [32P]dCTP using the Ready-to-Go DNA-labeling beads from Amersham Biosciences. Internal fragments of the *rot* gene (amplified with the primers o-loj-rot-7 and o-loj-rot-10), *spa* (amplified with the primers described in [20]) or the *rnalll* gene (amplified with the primers 5'- GATCACAGAGATGTTATGG + 5'-CATAGCACTGAGTCCAAGG) were used as template in the labeling reactions. All steps were repeated in three independent experiments giving similar results.

### Measurements of β-galactosidase activity

Cells were grown in TSB as described above to the indicated OD_600_. 1 ml aliquots were harvested and immediately frozen and stored at −20°C. Cell pellets were resuspended in 300 µl 0.9% NaCl containing 5 µg/ml lysostaphin and incubated at 37°C for 15 min until the samples became clear indicating lysis was complete. Cellular debris was removed by centrifugation and the samples were transferred to fresh tubes. The protein concentration of each sample was measured using the Bradford dye-binding procedure from Bio-Rad. Expression of β-galactosidase was quantified by adding 400 µl Z-buffer contaning 1 mg/ml ONPG to 100 µl of each sample as described previously [36]. Samples were incubated at 37°C until the solution had become yellow or not longer than 180 min. The reaction was stopped by adding 500 µl 1 M NaCO_3_ and β-galactosidase activity was measured at OD_420_ and the specific activity (1 unit  = 1 nmole ο-nitrophenol produced per min per mg protein) was calculated. All steps were repeated in three independent experiments giving similar results.

#### Statistical analysis

All significant values were determined using the 2 sample t-test with unequal variances. Error bars represent standard deviations.

## Results

### Cells lacking ClpX contain reduced levels of the Rot transcriptional regulator

**Figure 2:**
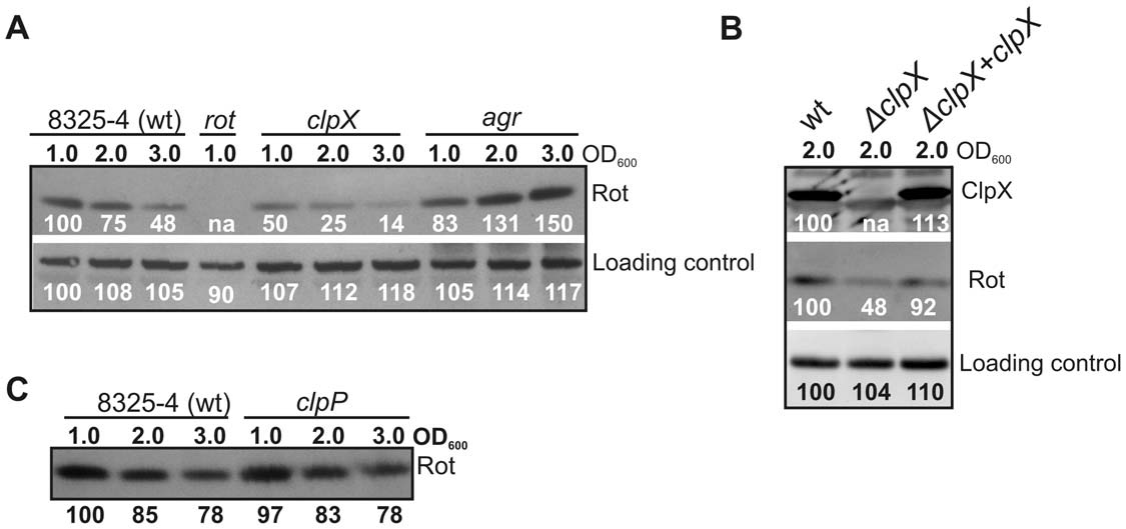
Expression of Rot is decreased in cells lacking ClpX. Proteins from wild-type, and *clpX, rot,* or *agr* mutant cells, (A), wt, *clpX* mutant cells and *clpX* complemented *clpX* mutant cells (Δ*clpX*+*clpX*) (B), and wild-type and *clpP* mutant cells (C) were extracted from exponential (OD_600_ = 1.0 (+/−0.1)) or post-exponential cells (OD_600_ = 2.0 (+/−0.1) and OD_600_ = 3.0 (+/−0.1) as indicated. In each blot extracted proteins were separated by SDS-PAGE, blotted onto a PVDF membrane and probed with Rot antibody (A, B, central panel and, C) and ClpX antibody (B, upper panel). To visualize equal loading, signals detected from non-specific binding of the Rot antibody to an unknown cellular protein are shown. The Rot protein levels in (A) and (C) were quantified relative to the wild-type Rot level in exponential cells (100%) using NIH imageJ 1.40. The ClpX protein, Rot protein and loading control protein levels in (B) were quantified relative to the wt level (100%) using Gene-snap from Perkin Elmer. The Western blots were repeated in at least 4 independent experiments giving similar results.

Similar to the transcriptional regulator Rot, ClpX is required for transcription of the *spa* gene, encoding Protein A [20,21]. Based on this observation we proposed that the chaperone activity of ClpX is required either to activate Rot, or, to control expression of Rot [20]. To estimate the level of Rot protein in the *clpX* mutant cells, we prepared polyclonal antibodies against Rot. The Rot antibodies were used to determine the relative amount of Rot protein in wild-type cells and in cells carrying deletions in *clpX*, *clpP,* or *agr*. As translation of the *rot* mRNA is inhibited by RNAIII [17], the Rot level was assayed both in exponential growth phase (prior to induction of RNAIII) and in post-exponential growth phase (RNAIII induced) (Fig. 2). The anti-Rot antibodies recognized a protein of the expected 16 kDa that was absent in protein extracts derived from the *rot* mutant (Fig. 2A). In wild-type cells the Rot protein was most abundant during exponential growth phase (Fig. 2A), which is consistent with the inhibitory role of RNAIII on *rot* translation [17,18]. In post-exponential cells the Rot level was gradually reduced, reaching 75% and 50% of the exponential level at OD_600_ = 2.0 and 3.0, respectively. In accordance with the proposed model, the down regulation of Rot was clearly dependent on *agr*. Interestingly, cells lacking ClpX contained 2–3 fold less Rot protein than wild type-cells at all assayed time-points (Fig. 2A). As restoration of the chromosomal *clpX* locus, resulted in wild-type expression of both Rot and Protein A we conclude that ClpX indeed has a stimulatory effect on Rot synthesis (Fig. 2B and data not shown).

The post-exponential down regulation of Rot was still observed in the *clpX* mutant, indicating that the effect of ClpX and RNAIII on Rot expression is mediated by different pathways. Moreover, cells lacking *clpP* contained wild-type levels of Rot protein at all assayed time-points (Fig. 2C), signifying that ClpX enhances Rot expression independently of ClpP.

### Expression of *spa* is activated above a threshold level of Rot

**Figure 3:**
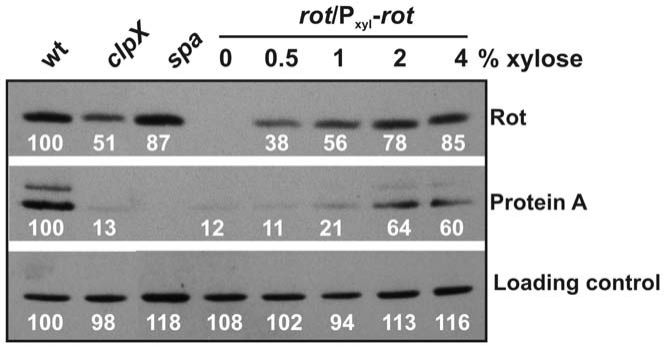
Rot performs threshold regulation of Protein A expression. The effect of the Rot concentration on Protein expression A was assayed by growing a *rot*-mutant expressing *rot* from a xylose inducible promotor in five parallel cultures supplemented with increasing amounts of xylose. Samples were collected at OD_600_ = 1.0 and total protein was extracted, equal amounts of protein in each lane was separated by SDS-PAGE, blotted onto a PVDF membrane, and probed to Rot polyclonal antibody (upper panel) and Protein A monoclonal antibody (lower panel). For comparisons, the Rot- and Protein A levels were determined in wild-type cells and in cells lacking either *clpX* or *spa.* To visualize equal loading, signals detected from non-specific binding of the Rot antibody to an unknown cellular protein are shown in the bottom panel. The Rot protein, Protein A, and loading control protein levels were quantified relative to the respective wild-type level in exponential cells (100%) using NIH imageJ 1.40. Each experiment was performed three times with similar results.

After confirming that ClpX is required for full expression of Rot, we examined whether the 2–3 fold reduction of the Rot level can explain the dramatically reduced Protein A level of the *clpX* mutant [20]. To control the Rot-level, we employed a *rot* mutant that harbours a plasmid carrying the *rot* gene transcribed from a xylose inducible promoter [11]. Protein samples were derived from exponential cultures grown with varying amounts of xylose, and were used for Western blot detection of Rot and Protein A. As seen in Fig. 3, upper panel, Rot expression increased with increasing concentrations of xylose, and with 4% added xylose the Rot level approached the wild-type level. The experiment clearly demonstrated that Protein A synthesis was dependent on the Rot levels (Fig. 3, central panel). Specifically, Protein A expression remained low until the level of Rot protein constituted approximately 75% of the wild-type level. As Rot controls Protein A synthesis at the level of transcription, this finding indicates that a threshold concentration of Rot must be exceeded to obtain induction of *spa* transcription. Importantly, in the *clpX* mutant, the Rot level was below this threshold level (the level was comparable to the Rot level in cells grown in the presence of 0.5–1% xylose). Hence, this experiment confirms that the reduced level of Rot in the *clpX* mutant can explain the severely reduced *spa* transcription in this strain [20]. Additionally, our results suggest that Protein A expression is regulated by a threshold mechanism.

### The essential roles of ClpX and Rot in Protein A expression are conserved in a clinical strain

**Figure 4:**
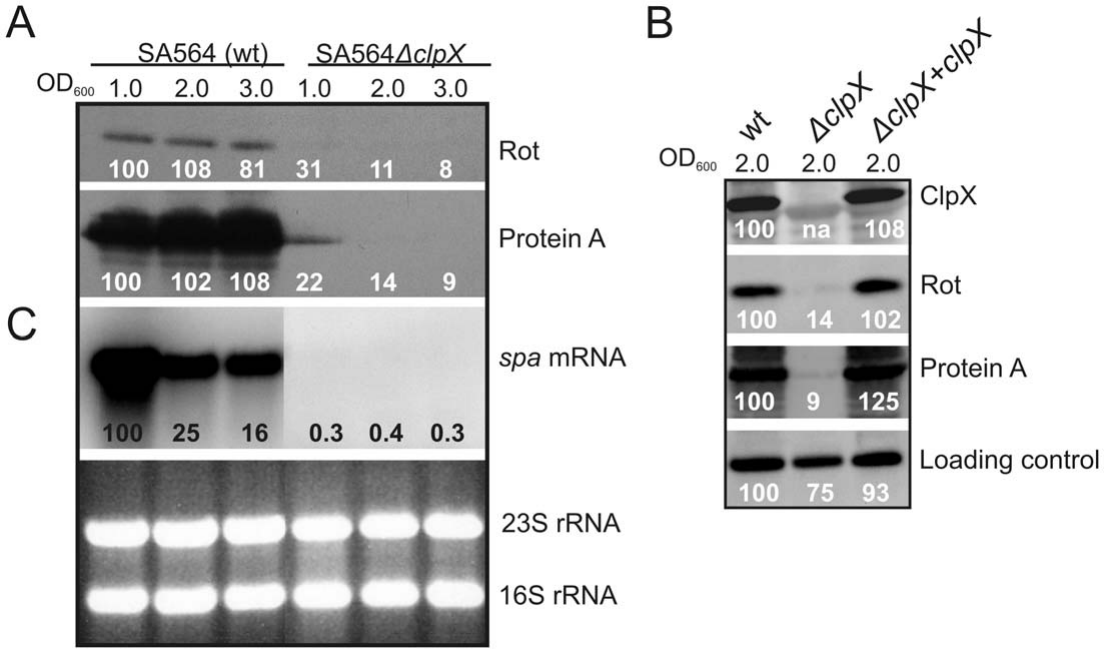
Protein A and Rot expression is substantially reduced in a clinical isolate carrying the *clpX* deletion. (A) Rot antibody and, Protein A antibody, were used to estimate the Rot, and Protein A levels in SA564 and the *clpX* deletion mutant at the indicated time-points. (B) ClpX antibody, Rot antibody, and Protein A antibody were used to estimate the ClpX, Rot, and Protein A levels in post-exponential cells (OD_600_ = 2.0 (+/−0.1)) of SA564, the *clpX* deletion mutant derived from it, and the *clpX* complemented *clpX* mutant (Δ*clpX*+*clpX*). To visualize equal loading the signals detected from non-specific binding of the Rot antibody to an unknown cellular protein is shown. Rot protein and Protein A levels were quantified relative to the wild-type Rot/Protein A level in exponential cells (100%) using Gene Snap from Perkin Elmer. (C) At the same time-points total RNA was extracted and the level of *spa* transcript in SA564 wild-type and *clpX* mutant cells was assessed by Northern blot analysis (third panel). The *spa* signals were quantified relative to the wild-type SA564 *spa* signal in exponential cells ( = 100%) using the Cyclone Plus Phosphor imager from PerkinElmer. To visualize equal loading, a picture of the ethidium stained agarose gel is shown in the bottom panel. Note that the middle 5 lanes containing samples not relevant to this study have been removed from both the Northern blot and the agarose gel.

The increasing focus on virulence gene regulation in clinical strains has emphasized that regulatory models established in laboratory strains may not be representative of the regulatory mechanisms observed in clinical isolates [37–39]. To assess if the role of ClpX in Rot and Protein A synthesis is conserved in clinical isolates, we made the 651 bp in-fame deletion of the *clpX* gene in the low-passage isolate SA564 derived from a patient with toxic shock syndrome [37]. Expression of Protein A and Rot in the SA564Δ*clpX* strain and SA564 wild type strain was compared using Western blot analysis (Fig. 4A). Strikingly, the stimulatory role of ClpX on Rot/ProteinA synthesis appears even more pronounced in the SA564 background, as deletion of *clpX* in SA564 substantially reduced (approximately 6 fold) the cellular level of Rot, and concomitantly decreased *spa* transcription and Protein A synthesis to less than 5% of the wild-type level (Fig. 4A). Upon complementation of the *clpX* locus, Protein A and the Rot level were returned to wild-type levels (Fig. 4B). We conclude, that the positive roles of ClpX on Rot and Protein A synthesis are conserved in a low-passage clinical strain, suggesting that the underlying mechanism is conserved among *S. aureus* strains.

**Figure 5:**
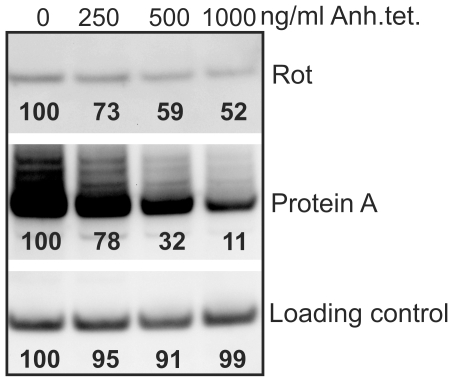
Protein A expression is dependent on the Rot level in SA564. To reduce the level of Rot in SA564, anti-sense *rot* mRNA was expressed from the *tetO* promoter in pRMC2 in the presence of increasing concentrations of the inducer anhydrotetracyclin. Anhydrotetracyclin was added at OD_600_ = 0.4 and proteins were extracted from samples derived at OD_600_ = 2.0. Western blotting was performed with Rot antibody (upper panel) and protein A mono-clonal antibody (second panel). To visualize equal loading the signals detected from non-specific binding of the Rot antibody to an unknown cellular protein is shown. All protein levels were quantified relatively to the corresponding signal obtained in the un-induced culture (100%) using Gene Snap from Perkin Elmer.

The SA564 strain expresses considerably more Protein A than 8325-4, and we therefore examined if Protein A expression is dependent on a threshold-level of Rot, as was observed for 8325-4 (Fig. 3). To reduce the cellular Rot level in SA564, the *rot* gene was cloned in the anti-sense orientation under control of the *tetO* promoter in the expression vector pRMC2 (see Materials and Methods for details; 32). As can be seen in Fig. 5, we could achieve a minor reduction (2 fold at the highest concentration of anhydrotetracycline) in the cellular Rot concentration by inducing transcription of the *rot* anti-sense mRNA. Interestingly, this modest reduction of Rot reduced expression of Protein A as much as 10 fold. This finding supports that below a threshold level of Rot, Protein A synthesis is greatly reduced.

### rot transcription is slightly reduced in *clpX* and *clpP* mutant cells

**Figure 6:**
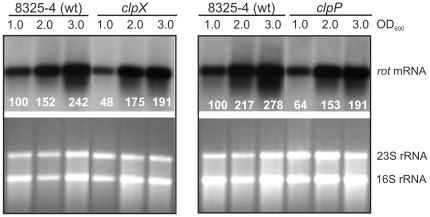
Expression of *rot* transcript is slightly decreased in *clpX*, and *clpP* mutant cells. The level of *rot* transcript was estimated by Northern blot analysis: Total RNA was extracted from wild-type and mutants lacking either *clpP* or *clpX* and separated on an agarose gel. Equal amounts of RNA were loaded in each lane. The RNA was blotted onto a nitrocellulose membrane and probed with a *rot* specific probe. Upper panel shows the resulting Northern blot, while ethidium stained agarose gel is shown below. The result is representative of 3 independent experiments.

We next attempted to pin-point the level at which ClpX mediates its affect on Rot synthesis. We first examined if ClpX controls Rot synthesis at the level of transcription. To this end, Northern blot analysis was performed to determine the level of *rot* transcript in exponential and post-exponential wild-type, *clpP*, and *clpX* mutant cells (Fig. 6). The Northern blot revealed that both *clpX* and *clpP* mutant cells contained *rot*-transcript levels that were slightly reduced compared to wild-type levels. As described above, the Rot protein level in the *clpP* mutant strain was comparable to the wild-type level, suggesting that the reduced *rot* transcription in *clpX* and *clpP* mutant strains is not reflected at the protein level. The Northern blot also revealed that in all strains transcription of *rot* increased in the post-exponential growth phase, as also reported by others [18]. The post-exponential induction of *rot* transcription occurs concomitantly with the down-regulation of Rot at the protein level emphasizing the significance of posttranscriptional regulation in Rot expression. From this experiment, we conclude that ClpX controls Rot synthesis mainly at the post-transcriptional level.

### ClpX does not control Rot expression by stimulating transcription of RNAIII

**Figure 7:**
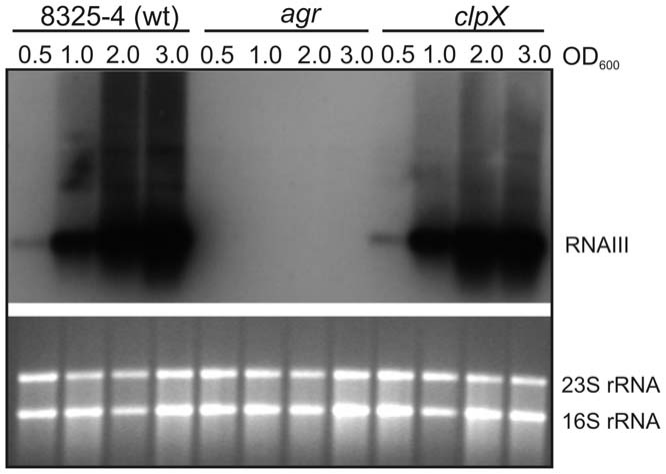
Expression of RNAIII is not altered in the *clpX* mutant. Cells of the wild-type and the *agr*, and *clpX* mutants were grown in TSB and at the indicated optical densities total RNA was extracted and separated on an agarose gel. RNA was blotted onto a nitrocellulose membrane and probed with an RNAIII specific probe. Below the blot is a picture of the ethidium stained agarose gel. The blot presented is representative of three independent experiments.

Next, we addressed if ClpX controls Rot expression by enhancing expression of RNAIII that is an inhibitor of Rot translation [17]. To this notion, the level of RNAIII in wild-type, *agr,* and *clpX* mutant cells in exponential and post-exponential growth phase was determined by Northern blot analysis (Fig. 7). The experiment demonstrated that the RNAIII level in the *clpX* mutant does not deviate from the wild-type levels. Hence, we ruled out that the reduced Rot level in the *clpX* mutant is accomplished through an increase in RNAIII synthesis.

### ClpX stimulates translation of *rot* mRNA

**Figure 8:**
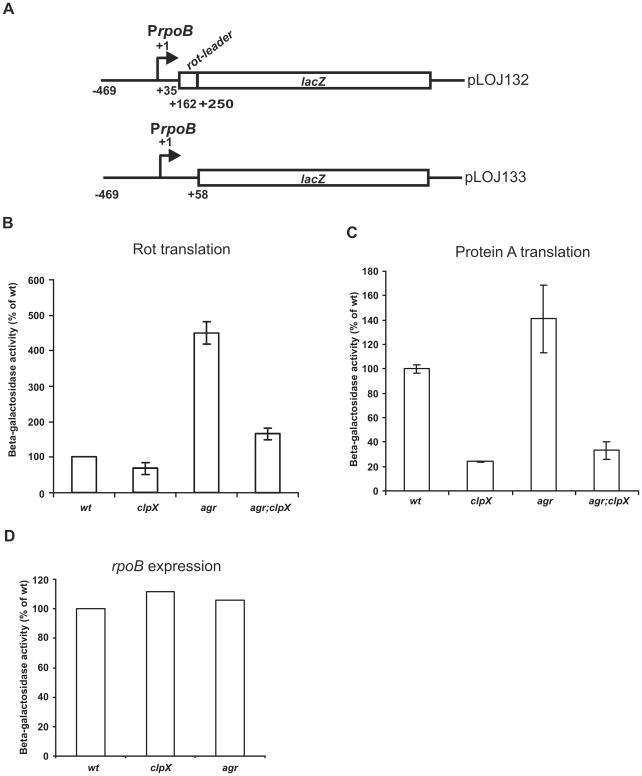
Translation of Rot and Protein A is decreased in the absence of ClpX. (**A**) Schematic drawing of the *lacZ* translational fusion points in pLOJ132 and pLOJ133. pLOJ132 is a P_rpoB_-*rot*-*lacZ* translational fusion. Transcription is driven from the constitutive P_rpoB_ promoter. The *rot*-leader encompassing the RNAIIII interacting region from +162 to +250 (numbered with respect to the transcriptional start site) is translationally fused to the *lacZ* gene. pLOJ133 is a P_rpoB_-*lacZ* translational fusion. (**B**) β-Galactosidase activity measured in post-exponential (OD_600_ = 2.0) wild-type- and, *clpX*, *agr*, *agr;clpX* mutant strains harbouring pLOJ133. The specific activities are presented as a percentage of the wild-type activity (100%). P-values were calculated using the two-sample t-test with unequal variances: wild-type different than clpX: P-value = 0.008; agr different than agr;clpX: P-value = 0.0004. The statistical calculations were based on five independent experiments. (**C**) Protein A translation was measured as β-Galactosidase activity expressed from pLUG520 in wild-type- and *clpX*, *agr*, and *agr;clpX* mutant cells in the post-exponential growth phase (OD_600_ = 2.0). Specific activities are presented relative to the wild-type level (100%). (**D**) β-Galactosidase activity measured in post-exponential (OD_600_ = 2.0) wild-type and mutant cells that harbour pLOJ133 carrying a translational fusion between *rpoB* and *lacZ*. The specific activities have been calculated relative to the wild-type level (100%). Each experiment was performed at least four independent times with similar results.

To investigate directly whether ClpX regulates Rot translation, a translational fusion between the 5′-*rot*-leader and *lacZ* was constructed as depicted in Fig. 8A. This construct encompasses the RNAIII interacting region, the ribosome binding site, and the first three amino acid codons of the *rot* leader translationally fused to *lacZ* [17,33]. Transcription of the fusion is driven by the constitutive promoter P_rpoB_. The plasmid harbouring the *rot*-translational fusion was introduced into wild-type, and *agr*, *clpX*, *clpP*, and *agr;clpX* mutant strains, and β-galactosidase activity was assayed in samples derived from post-exponential cultures (Fig. 8B). The assay revealed that β-galactosidase activity was almost five fold higher in the *agr* mutant than in the wild-type (Fig. 8B). This suppressive effect of RNAIII on *rot*-translation is in the same range as observed by others and, thus, validates the constructs [17]. While samples derived from *clpP* mutant cells expressed wild-type levels of β-galactosidase (data not shown), β-galactosidase activity was reduced approximately two fold compared to wild-type in *clpX* mutant cells. This two-fold difference was statistically significant (wild-type different from *clpX*; P-value = 0.008), indicating that ClpX indeed has a positive effect on translation of *rot*. Interestingly, β-galactosidase activity in the *agr*, *clpX* double mutant was substantially reduced compared to the activity measured in the *agr* single mutant strain. This suggests that even though the inhibitory effect of RNAIII is relieved by a deletion of the *agr* locus, translation of Rot still depends on ClpX. Taken together; these results specify that ClpX stimulates *rot* translation, and that the role of ClpX in *rot* translation is not linked to the RNAIII mediated inhibition of *rot* translation.

### ClpX stimulates translation of *spa* mRNA but not of *rpoB* mRNA

Huntzinger *et a*l. showed that translation of *spa* (Protein A), similar to translation of Rot, is inhibited by RNAIII [12]. To examine if ClpX has a role in translation of Protein A, the plasmid, pLUG520, carrying the P_rpoB_-*spa*-*lacZ* translational fusion used by Huntzinger *et al*. [12] was introduced into wild-type and mutant strains. This construct encompasses the constitutive *rpoB* promoter and the first 63 nucleotides of the *spa* mRNA (including the sites for RNAIII interaction) fused in frame with *lacZ* deleted of its ribosomal binding site, and thus specifically measures the translational activity of the *spa*-leader. In samples derived from post-exponential cultures, β-galactosidase activity was approximately 1.5 fold higher in the *agr* background than in the wild-type background, in accordance with previous results obtained by Huntzinger *et a*l. [12] (Fig. 8C). Importantly, the absence of ClpX reduced β-galactosidase activity expressed from the *spa-lacZ* translational fusion approximately 5 fold, both in wild-type and in cells devoid of *agr*/RNAIII. We conclude that translation of *spa* mRNA, similar to translation of *rot* mRNA, is positively affected by ClpX and that the positive effect of ClpX on *spa* and *rot* translation occurs independently of RNAIII.

To investigate if ClpX also contributes to translation of mRNAs not interacting with RNAIII, we examined expression of the house-keeping gene *rpoB* in the *clpX* mutant by using a translational fusion between the *rpoB* promoter and *lacZ* (pLOJ133). With this plasmid, β-galactosidase activity was similar in samples derived from all strains demonstrating that neither RNAIII nor ClpX regulates expression of RpoB (Fig. 8D).

## Discussion

Protein A is an abundant surface protein that by its ability to bind the Fc fragment of immunoglobulins from mammalian species is believed to be important for immune evasion of *S. aureus* [5]. Previously, we have shown that ClpX (but not ClpP), similar to the transcriptional regulator Rot, is essential for transcription of the *spa* gene encoding Protein A, and therefore we hypothesized that the chaperone activity of ClpX is required for either synthesis or activity of Rot [20]. In the present study we confirm that ClpX is indeed required for full expression of Rot, as cells devoid of ClpX contain approximately 2–3 fold less Rot protein than wild-type cells. This finding raised the question whether the relatively modest 2–3 fold reduction in the cellular Rot level is sufficient to abolish *spa* transcription. We confirmed this by varying Rot expression from an inducible promoter and demonstrated that a 2 fold reduction in Rot expression causes a dramatic 90% reduction in Protein A expression in both 8325-4 and the low-passage clinical isolate SA564 [40]. This finding emphasizes that major changes in Protein A expression may be achieved by small reductions in the Rot level and suggests that a threshold level of Rot is needed for synthesis of protein A.

Rot stimulates Protein A transcription both directly, by binding to the *spa* promoter, and in-directly by enhancing transcription of *sarS*, encoding another positive activator of *spa* transcription [11,13,41]. Interestingly, transcription from the *spa* promoter was suggested to be regulated by negative (SarA) and positive regulators (SarS and Rot) competing for overlapping operator sites [13]. Therefore we propose that the threshold level of Rot (and SarS) is required to relieve the repression imposed by SarA, and that the ClpX dependent expression of Rot is required to exceed this threshold level. It is tempting to speculate that additional genes of the Rot regulon are subject to a similar regulatory mechanism.

As deletion of *clpX* severely decreased the Rot level (6 fold compared to the max 3-fold in 8325-4), and accordingly abolished expression of Protein A in the low-passage isolate SA564, we propose that the mechanism by which ClpX controls the Rot and Protein A level is conserved among clinical *S. aureus* isolates.

Attempts to pin-point the level at which ClpX mediates its effect on Rot synthesis demonstrated that ClpX stimulates expression of Rot both at the level of transcription and translation (Fig. 6 and 8). Based on the finding that transcription of *rot* was decreased similarly by the absence of either ClpX or ClpP, we propose that ClpX controls *rot* transcription as part of the ClpXP proteolytic complex. Notably, the *clpP* mutant strain contained wild-type levels of Rot suggesting that the reduction in *rot* transcription observed in *clpX* and *clpP* mutant strains is not reflected in the cellular Rot level, and hence that the ClpP independent effect of ClpX in controlling Rot synthesis is achieved mainly at the post-transcriptional level. Accordingly, we were able to show that ClpX does indeed stimulate translation of the *rot* mRNA, and that ClpX additionally enhances translation of the *spa* mRNA. Initially this observation prompted us to suspect that ClpX modulates the interaction between RNAIII and its target mRNA's. This interaction involves base pairings between RNAIII and secondary hair-pin structures in the target mRNA by which dual loop-loop complexes are formed. These interactions block ribosomal access to the Shine-Dalgarno of the respective messengers, thereby inhibiting translation [17,18]. However, as we observed a substantial ClpX dependent stimulation of *spa* and *rot* translation in cells lacking RNAIII, we conclude that ClpX stimulates translation independently of RNAIII.

Secondary structure determinations of the *spa*- and *rot*-mRNAs reveal that the translational signals are partially obscured in the stem-loop structures, also when not bound to RNAIII [12,17]. Consequently, it is possible that the structures of the *spa* and *rot* transcripts required for interactions with RNAIII could hamper interactions with the ribosomes even in the absence of RNAIII. Hence, one way ClpX may promote translation of the *rot* and *spa* mRNAs is by, directly or indirectly, unfolding the stem-loop structures to facilitate interaction of these mRNAs with the translational apparatus.

In *E. coli*, the abundant RNA chaperone Hfq has a central role in translational stimulation of specific mRNAs [42]. Hfq is the only RNA chaperone yet identified in *S. aureus*. Accordingly, Hfq could be a candidate through which ClpX mediates its function in translational stimulation. However, recently the biological significance of Hfq in *S. aureus* was questioned by the lack of phenotypes of an *hfq* mutant and by the lack of detection of the *hfq* mRNA [18,40]. Consistent with this finding we were not able to detect Hfq in our strains when performing Western blot analysis using anti-Hfq polyclonal antibodies (raised against Hfq purified from *S. aureus*, see Materials and Methods, Supplemental Fig. S1). Furthermore, as both expression of Protein A and Rot did not change in strains lacking Hfq [40], it seems unlikely that the stimulatory effect of ClpX on translation of the *rot*- and *spa* transcripts is mediated through Hfq. Rather, *S. aureus* may harbour a yet unidentified RNA chaperone that is subject to regulation by ClpX.

An intriguing alternative possibility is that ClpX could affect translation through direct binding to specific mRNAs. The ClpX family of proteins contains a C4 zinc-binding domain in the N-terminal region and this type of domain has been implicated in nucleic acid binding in both eukaryotic and prokaryotic organisms [43,44]. In addition, a eukaryotic relative of ClpX, the HSP101 heat shock protein of plants specifically binds to and stimulates translation of the A-rich 5′ un-translated leader of the tobacco mosaic virus [45]. Inspection of the three-dimensional structure of ClpX, furthermore, reveals a striking resemblance to the RNA binding protein Hfq as both proteins form homo-hexameric ring-structures with similar sizes of the central pores. Further experiments are underway to determine the mechanisms of ClpX mediated translational stimulation.

In conclusion, we have shown that ClpX has a dual role in stimulating Protein A synthesis, as ClpX enhances both transcription (through enhancing synthesis of Rot), and translation of *spa*. Our findings emphasize that ClpX through its role in fine-tuning the level of the central regulator Rot holds an important role in controlling global expression of virulence factors in *S. aureus*.

## Supporting Information

Figure S1 Hfq could not be detected in Western blot analysis. Cells were harvested in early stationary phase or late stationary phase. Cells were lysed as described in materials and methods and total protein extract were separated on an SDS-gel. 10 µg protein was loaded (0.5 µg purified S. aureus Hfq protein).and samples were heated to 95°C for 20 min prior to loading. For comparison of protein sizes, the PageRulerTM Plus Prestained Protein Ladder (Fermentas) was included (not shown). The Hfq antibody specifically recognizes the monomeric and multimeric species of the purified Hfq protein, whereas no bands at corresponding sizes were observed in any of the cellular protein samples tested.
(2.49 MB TIF)
