# Supplementary figures and images for: The Chaperone ClpX Stimulates Expression of Staphylococcus aureus Protein A by Rot Dependent and Independent Pathways
Source: PLoS One. 2010 Sep 14;5(9):e12752. doi: 10.1371/journal.pone.0012752 (PMC2939077; doi:10.1371/journal.pone.0012752)

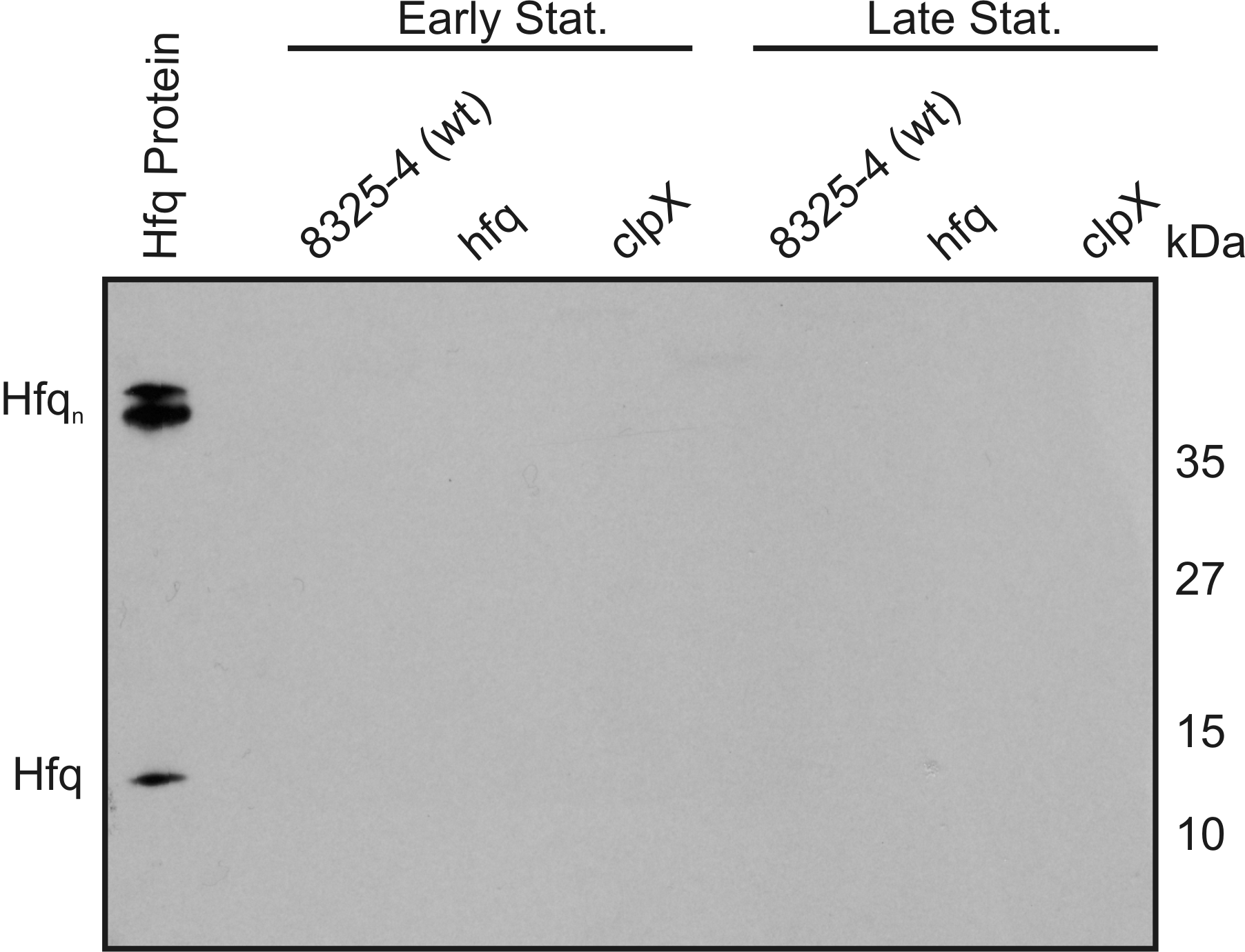

Supplement: Figure S1 — Hfq could not be detected in Western blot analysis. Cells were harvested in early stationary phase or late stationary phase. Cells were lysed as described in materials and methods and total protein extract were separated on an SDS-gel. 10 µg protein was loaded (0.5 µg purified S. aureus Hfq protein).and samples were heated to 95°C for 20 min prior to loading. For comparison of protein sizes, the PageRulerTM Plus Prestained Protein Ladder (Fermentas) was included (not shown). The Hfq antibody specifically recognizes the monomeric and multimeric species of the purified Hfq protein, whereas no bands at corresponding sizes were observed in any of the cellular protein samples tested. (2.49 MB TIF) [file pone.0012752.s001.tif]
